# Full-mouth rehabilitation of a patient with cleidocranial dysplasia using immediately loaded basal implant-supported fixed prostheses: A case report

**DOI:** 10.1016/j.ijscr.2019.11.005

**Published:** 2019-11-08

**Authors:** Abdelnasir G. Ahmad, Motaz Osman, Fadia Awadalkreem

**Affiliations:** a Oral and Maxillofacial Surgery Department, Khartoum Teaching Dental Hospital, Federal Ministry of Health, Khartoum, Sudan; b Implant Department, Khartoum Teaching Dental Hospital, Federal Ministry of Heath, Khartoum, Sudan; c Department of Oral Rehabilitation/Prosthodontic Division, Faculty of Dentistry, University of Khartoum, Sudan

**Keywords:** CCD, cleidocranial dysplasia, BCS, Basal Cortical Screw, Cleidocranial dysplasia, Basal implants, Implant-supported prostheses, Immediate loading, Case report

## Abstract

**Introduction:**

Cleidocranial dysplasia (CCD) is a rare congenital disease characterised by generalised skeletal and orofacial defects. The dental rehabilitation of patients with CCD is a case-sensitive procedure requiring a multidisciplinary approach.

**Presentation of case:**

A 24-year-old woman presented with the skeletal and oral manifestations of CCD, including multiple poorly erupted and impacted teeth. The patient was very depressed and rejected orthodontic treatment; therefore, we formulated a treatment plan involving extraction of all impacted and poorly erupted teeth under general anaesthesia, followed by fabrication and delivery of transitional complete dentures for cosmetic, functional, and psychological benefits. Three months later, 14 screw basal implants were inserted in the maxillary and mandibular jaws and subjected to immediate loading with fixed prostheses 3 days later. At the 3-year follow-up visit, the patient expressed satisfaction with the aesthetic and functional aspects of the prostheses and presented with excellent oral health.

**Discussion:**

The timing of diagnosis, patient’s age, willingness to undergo the planned treatment, and treatment duration are valuable considerations for establishing an appropriate treatment plan for the oral manifestations of CCD.

**Conclusion:**

To our knowledge, this is the first report on basal implant-based full-mouth rehabilitation in a patient with CCD. Basal implant-supported prostheses may be useful for patients with CCD who present with a limited bony foundation after tooth removal. The treatment requires lesser time than orthodontic treatment, eliminates the need for bone grafting, spares the patient from ill-ﬁtting dentures, reduces the overall cost, and improves the quality of life.

## Highlights

• A 24-year-old woman with cleidocranial dysplasia (CCD)refused orthodontic treatment.
• Fourteen screw basal implants were inserted in both jaws.
• The implants were immediately loaded 3 days later with fixed prostheses.
• The patient was satisfied and presented excellent oral health 3 years later.
• Basal-implant supported fixed prostheses can improve aesthetics and quality of life.

## 1 Introduction

Cleidocranial dysplasia (CCD) is a rare congenital disease with an autosomal dominant inheritance pattern and is characterised by generalised skeletal and orofacial manifestations [[1], [2], [3], [4]]. The disease may also arise because of genetic mutations, with 20 %–40 % cases diagnosed in the absence of any family history [1,4]. CCD has been mapped to chromosome 6 p21 [1,4], which is involved in the control of osteoblast differentiation and chondrocyte mutation during endochondral ossification.

The earliest known case of CCD was reported by Meckel in 1760 [2]. In 1765, Martin [2,5] reported a case of CCD with clavicular defects, while Scheuthauer reported a case characterised by a combination of clavicular and cranial defects in 1871 [2]. Subsequently, in 1897, Marie and Sainton [6] coined the term “cleidocranial dysostosis,” which was later changed to CCD.

The main features of CCD include the ability to approximate the shoulders anteriorly as a result of clavicular hypoplasia [1,2,4], frontal bossing [1], hypertelorism [1,7], a poorly developed midfrontal area, a low nasal bridge, reduced nasal length with increased nasal width and protrusion [1,7], and a short stature [1,2,7].

Cranial radiographs reveal late closure of the fontanelle, open skull sutures, and multiple Wormian bones, particularly in the coronal and lambdoid regions [2]. The thoracic cage is small and bell-shaped with short ribs. Pelvic radiographs show hypoplastic iliac wings, broad femoral necks with large epiphyses, and an unossified symphysis pubis.

Dental defects have been reported in 93.5 % patients with CCD. These include retention of deciduous teeth, delayed eruption of permanent teeth, multiple supernumerary teeth, and impacted teeth [[1], [2], [3],^7^].

Dental treatment planning varies among cases and primarily depends on the needs of the patient, age at diagnosis, and social and financial circumstances [8]. Nevertheless, the main objectives are to restore craniofacial and dental function, improve aesthetics, and maximise patient satisfaction [8].

The therapeutic approach to CCD has significantly improved. Initially, the condition was considered untreatable [3]. However, various treatment strategies have been developed for the oral manifestations, including the extraction of all teeth and their replacement by dentures [3,8] or a more conservative approach involving extraction of supernumerary and retained deciduous teeth with surgical exposure of impacted permanent teeth and orthodontically guided eruption [3]. In some cases, the impacted teeth are exposed, aligned, and used to support overdentures [4]. When orthodontic treatment is not possible or rejected by the patient, prosthetic rehabilitation may be the treatment of choice [3,8].

The use of conventional implants for supporting fixed or removable dental prostheses in patients with CCD has been documented [1,3,7]. However, some patients present with severe ridge resorption that can cause complications and prevent the use of conventional implants without ridge augmentation. Nowadays, basal implants are used as alternatives to conventional implants requiring bone augmentation. These implants are deeply anchored in the basal bone [[9], [10], [11], [12]], and their stability is primarily achieved by bicortical anchorage, which can be provided by the crestal cortex and the nasal sinus bone, tuberosity or pterygoid plates in the maxilla and the crestal bone, buccal and lingual plates, or crestal and inferior plates in the mandible. These characteristics make basal implants suitable for use in patients with CCD and severely resorbed ridges. Here we describe the full-mouth rehabilitation of a 24-year-old woman with CCD using immediately loaded basal implant-supported fixed prostheses.

This work has been reported in line with the SCARE criteria [13].

## 2 Presentation of case

**Fig. 1:**
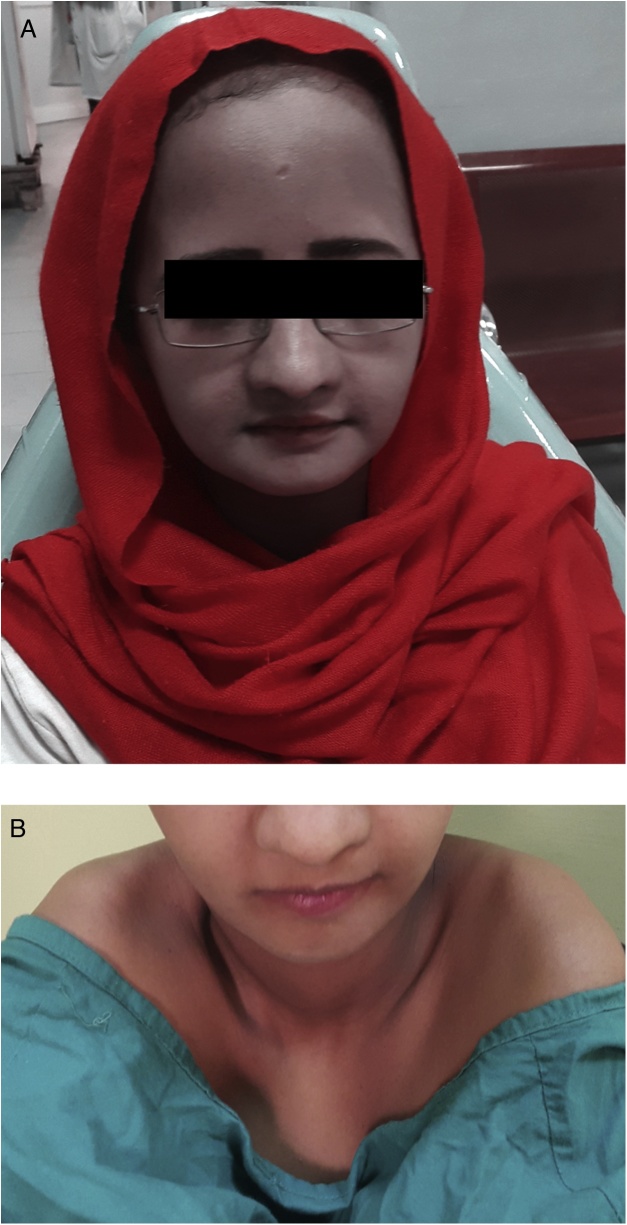
Preoperative clinical photographs of a 24-year-old woman with cleidocranial dysplasia. A. A frontal view shows frontal and parietal bossing, hypertelorism, and a depressed nasal bridge. B. The patient’s photograph shows anterior approximation of the shoulders because of the abnormal position of the clavicles.

**Fig. 2:**
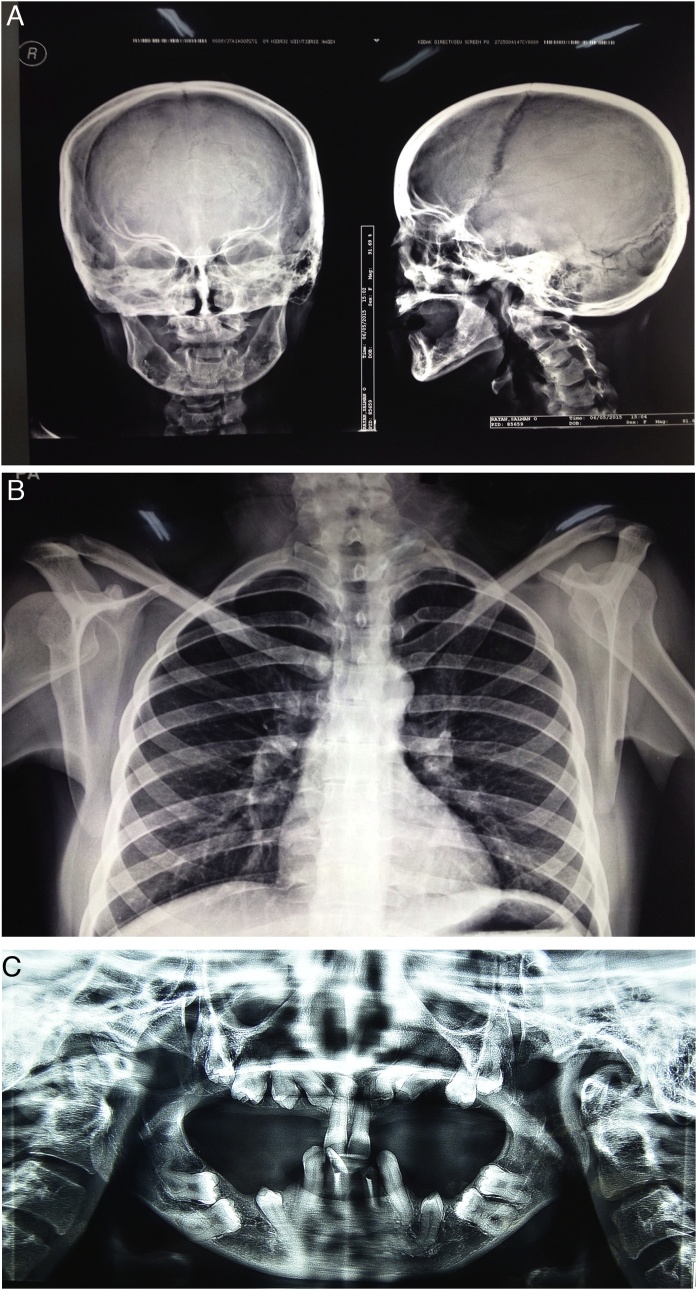
Preoperative radiographs of a 24-year-old woman with cleidocranial dysplasia. A. Anteroposterior and lateral skull views show small maxillary sinuses and thick cranial cortical bone with open sutures. B. A chest radiograph shows a bell-shaped thoracic cage and abnormally positioned clavicles. C. A panoramic radiograph shows multiple retained deciduous teeth, unerupted permanent teeth, and reduced maxillary bone height.

A 24-year-old woman with no relevant family history and was not taking any medication, was referred to Khartoum Teaching Dental Hospital to undergo dental rehabilitation for cosmetic and functional reasons (Fig. 1A). Her childhood dental history was complicated, with several deciduous teeth being retained and some being exfoliated beyond the expected time. Moreover, several permanent teeth were impacted, some of which had been surgically exposed to facilitate the eruption process. On physical examination, she exhibited abnormal sloping and anterior approximation of the shoulders (Fig. 1B), in addition to frontal and parietal bossing, hypertelorism (intercanthal distance: 40 mm), and a depressed nasal bridge. The lower, middle, and upper facial heights were 60, 70, and 70 mm, respectively. Intraoral examination revealed a partially dentate state, with decayed maxillary central and mandibular central and lateral incisors. A skull radiograph revealed small maxillary sinuses and thick cranial cortical bone with open sutures (Fig. 2A). A chest radiograph revealed a small, bell-shaped thoracic cage (Fig. 2B). Panoramic radiography (Planmeca ProMax, Finland) showed multiple impacted teeth in all quadrants and reduced maxillary bone height (Fig. 2C). These findings led to a diagnosis of CCD.

### 2.1 Treatment

A multidisciplinary team (Maxillofacial Surgeon and Prosthodontists) was formed, and all therapeutic options were discussed with the patient, who was not willing to undergo orthodontic treatment to upright her molar teeth for cosmetic, psychological, financial, and time-related considerations. She expressed her desire for fixed prostheses. Therefore, we formulated a treatment plan that involved extraction of all impacted, poorly erupted, and decayed teeth under general anaesthesia; the use of transitional acrylic complete dentures for 3 months for psychological reasons; and fabrication and delivery of basal implant-supported fixed prostheses. This treatment plan was fully discussed with the patient, who provided informed consent for treatment and publication of this report.

Under general anaesthesia, a flap was raised, and a surgical bur was used to remove the bone as needed. The teeth were extracted, and curettage was performed for the removal of excess soft tissue. The flap was sutured, and the patient was recalled for suture removal after 1 week. Following soft tissue healing (2 weeks), transitional acrylic complete dentures were fabricated and delivered.

**Fig. 3:**
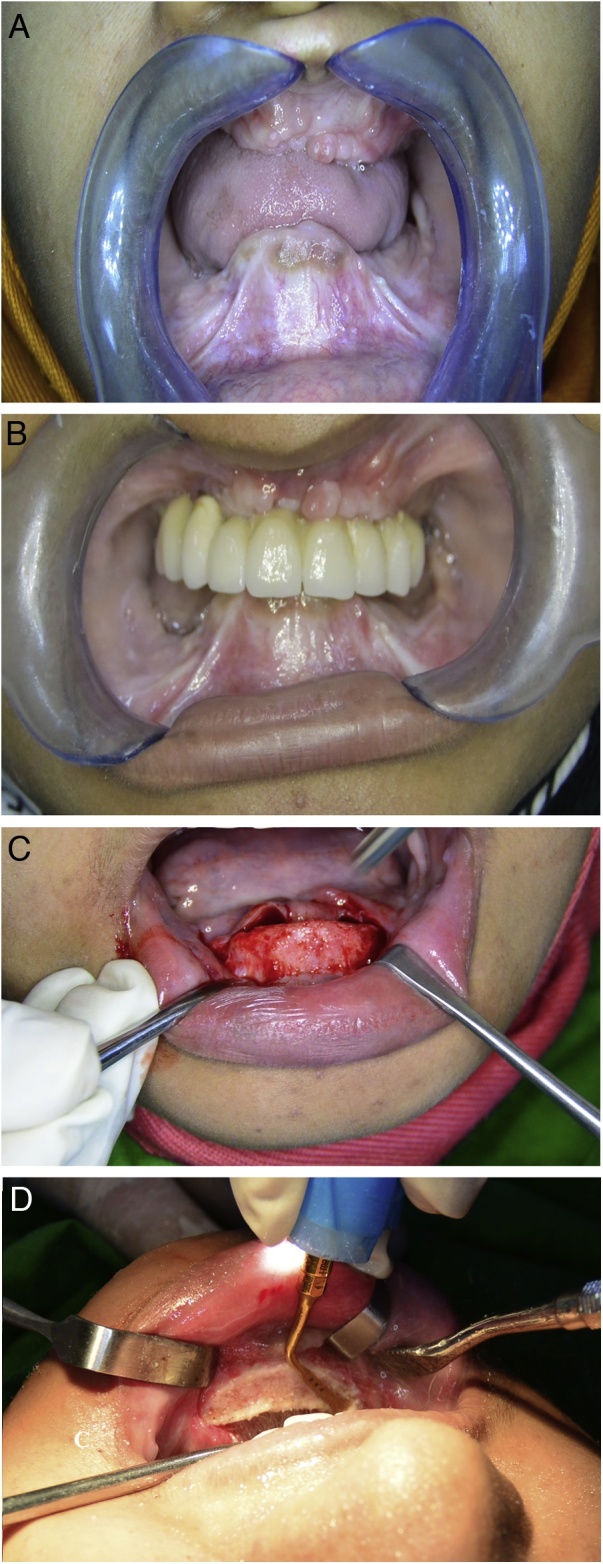
Intraoral rehabilitation of a 24-year-old woman with cleidocranial dysplasia using immediately loaded basal implant-supported fixed prostheses. A. Edentulous maxillary and mandibular ridges. B. the maxillary implant-supported prosthesis after insertion and cementation. C. Elevation of a periosteal flap in the mandibular anterior region. D. Alveolectomy using a piezosurgery device.

Three months later, the patient was recalled for implant placement. Both clinical and preoperative radiographs showed that the maxillary bone was extensively resorbed while the mandibular bone was resorbed in the posterior region and excessively protruding in the anterior region (Fig. 3A). Accordingly, we decided to use Basal Cortical Screw implants ([BCS®], Dr. Ihde Dental AG, Switzerland). Alveoplasty using a piezosurgery device (Mectron s.p.a., Italy) was performed in the mandibular anterior region.

In the maxilla, six BCS® implants were inserted using a flapless technique under local anaesthesia (2 % lidocaine with epinephrine 1:100,000). Impression copings were inserted and an impression was recorded using monophase silicone impression material (VPS; Ivoclar Vivadent AG). One day later, a metal framework was tried, and one days later, final ceramic prostheses were inserted and cemented using Fuji I glass ionomer luting cement (GC Corporation, Japan) (Fig. 3B).

**Fig. 4:**
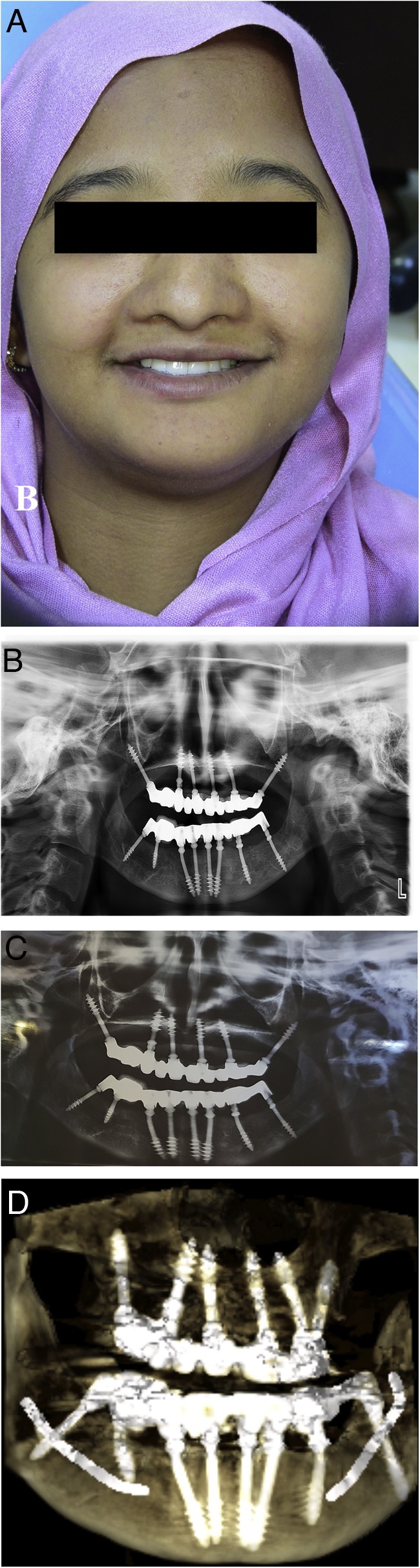
Postoperative and follow-up images of immediately loaded basal implant-supported fixed prostheses fabricated for a 24-year-old woman with cleidocranial dysplasia. A. A frontal view shows the inserted maxillary and mandibular basal-implant supported prostheses. B. A panoramic radiograph shows the maxillary and mandibular prostheses. C. A panoramic radiograph shows the maxillary and mandibular prostheses after 3 years of function. D. A three-dimensional cone beam computed tomography image shows the maxillary and mandibular prostheses after 3 years of function.

In the mandible, a flap was raised and alveolectomy followed by bone filling was performed (Fig. 3C,D). Eight BCS® implants were inserted and immediately loaded with fixed prostheses using the same procedure used for the maxilla (Fig. 4A). A postoperative panoramic radiograph and cone beam computed tomography images were acquired (Fig. 4B) The patient was provided with oral hygiene instructions, antibiotic, and recalled at 1 week; 3, 6, 9, 12, and 18 months; and every 6 months thereafter.

The patient had no complaints during the follow-up period and reported improvements in speech, mastication, and aesthetics. Overall, she was satisfied with her treatment. After 3 years, she exhibited optimal peri-implant health without any implant-related or prosthetic complications (Fig. 4C,D).

## 3 Discussion

CCD is a congenital disease with several skeletal and dental features. Our patient presented with almost all the diagnostic features of CCD described in the literature [[1], [2], [3], [4]]. Dental rehabilitation of patients with CCD is a challenge and requires a multidisciplinary team approach with good collaboration between an expert surgeon and a prosthodontic team [4].

The timing of diagnosis, patient’s age and willingness to receive the planned treatment, and treatment duration are valuable considerations for establishing an appropriate treatment plan for the oral manifestations of CCD. Our patient was 24 years old and extremely depressed. She rejected orthodontic treatment and bone grafting procedures and desired an immediate solution for her dental problems. Accordingly, we decided to extract all teeth and deliver implant-supported fixed prostheses in order to prevent further bone resorption and provide comfortable, stable prostheses [3,8].

According to previous reports, the use of basal implants enables the fabrication of fixed prostheses for severely resorbed edentulous arches and facilitates immediate functional loading [[9], [10], [11], [12]]. Thus, we believed that basal implant-supported prostheses were the best treatment options for our patient, who exhibited a very limited bony foundation after tooth removal. This treatment was not as prolonged as orthodontic treatment, eliminated the need for bone grafting procedures, spared the patient from ill-ﬁtting dentures, reduced the overall cost, and improved the patient’s quality of life by restoring aesthetics, speech, and function.

Despite the fact that genetic mutations compromise osteoblastic activity in patients with CCD, evidence of bone remodelling and osseointegration has been reported [1,3,7,8]. Even in the present case, the patient presented with a stable, well-functioning prosthesis without biological (optimal peri-implant soft tissue health), aesthetic, functional, or mechanical complications at 3 years after treatment completion. In addition, her overall satisfaction level was high.

## 4 Conclusion

To our knowledge, this is the first report on basal implant-based full-mouth rehabilitation in a patient with CCD. For adult patients with CCD who refuse orthodontic treatment, extraction of impacted and supernumerary teeth followed by the fabrication and delivery of immediately loaded basal implant-supported fixed prostheses could be a valuable treatment option that markedly improves aesthetics, speech, function, and the overall quality of life.

## Sources of funding

No funding was obtained for this study.

## Ethical approval

Ethical approval was obtained from the research ethical committee of Khartoum Dental Teaching Hospital.

## Consent

Written informed consent was obtained from the patient for publication of this case report and supplementary images.

## Author’s contribution

All the authors contribute in the treatment of the patient, writing and finalization of the manuscript.

## Registration of research studies

The research was registered at the research centre of the Khartoum Dental Teaching Hospital, Federal Ministry of Health, Khartoum, Sudan after approval by the research ethical committee of Khartoum dental teaching hospital.

## Guarantor

Dr. Abdelnasir G Ahmad.

## Provenance and peer review

Not commissioned, externally peer-reviewed.

## Declaration of Competing Interest

The authors have no conflict of interests.
